# 2-(2,3-Difluoro­phen­yl)ethyl toluene-4-sulfonate

**DOI:** 10.1107/S1600536809055329

**Published:** 2010-01-09

**Authors:** Xiaoqiang Sun, Chunyan Shao, Yuan Cui, Xiuqin Zhang, Rongqing Lu

**Affiliations:** a Key Laboratory of Fine Petrochemical Engineering, Jiangsu Polytechnic University, Changzhou 213162, People’s Republic of China; b High Technology Research Institute of Nanjing University, Changzhou 213162, People’s Republic of China

## Abstract

In the title compound, C_15_H_14_F_2_O_3_S, the dihedral angle between the aromatic rings is 6.19 (13)°. In the crystal, mol­ecules are linked by C—H⋯O hydrogen bonds, generating [110] chains.

## Related literature

For related structures, see: Zhang & Zang (2008 ▶); Xi *et al.* (2008 ▶); Wang & Qin (2008 ▶). 
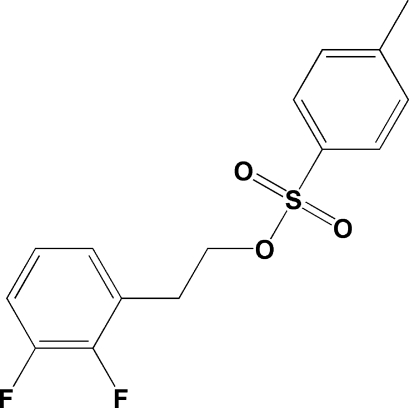


## Experimental

### 

#### Crystal data

C_15_H_14_F_2_O_3_S
*M* *_r_* = 312.32
Triclinic, 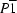

*a* = 7.487 (12) Å
*b* = 8.386 (14) Å
*c* = 12.69 (2) Å
α = 91.67 (3)°
β = 96.51 (3)°
γ = 105.65 (3)°
*V* = 761 (2) Å^3^
*Z* = 2
Mo *K*α radiation
μ = 0.24 mm^−1^
*T* = 295 K
0.21 × 0.21 × 0.16 mm

#### Data collection

Bruker APEXII CCD diffractometer
Absorption correction: multi-scan (*SADABS*; Bruker, 2003 ▶) *T*_min_ = 0.955, *T*_max_ = 0.966
4133 measured reflections
2630 independent reflections
2246 reflections with *I* > 2σ(*I*)
*R*_int_ = 0.018

#### Refinement

*R*[*F* ^2^ > 2σ(*F* ^2^)] = 0.046
*wR*(*F* ^2^) = 0.180
*S* = 1.10
2630 reflections
191 parameters
H-atom parameters constrained
Δρ_max_ = 0.35 e Å^−3^
Δρ_min_ = −0.25 e Å^−3^

### 

Data collection: *APEX2* (Bruker, 2003 ▶); cell refinement: *SAINT* (Bruker, 2003 ▶); data reduction: *SAINT*; program(s) used to solve structure: *SHELXS97* (Sheldrick, 2008 ▶); program(s) used to refine structure: *SHELXL97* (Sheldrick, 2008 ▶); molecular graphics: *SHELXTL* (Sheldrick, 2008 ▶); software used to prepare material for publication: *SHELXTL*.

## Supplementary Material

Crystal structure: contains datablocks I, global. DOI: 10.1107/S1600536809055329/hb5283sup1.cif

Structure factors: contains datablocks I. DOI: 10.1107/S1600536809055329/hb5283Isup2.hkl

Additional supplementary materials:  crystallographic information; 3D view; checkCIF report

## Figures and Tables

**Table 1:** Hydrogen-bond geometry (Å, °)

*D*—H⋯*A*	*D*—H	H⋯*A*	*D*⋯*A*	*D*—H⋯*A*
C1—H1⋯O1^i^	0.93	2.58	3.442 (7)	154

Symmetry code: (i) 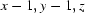.

**Fig. 1.**
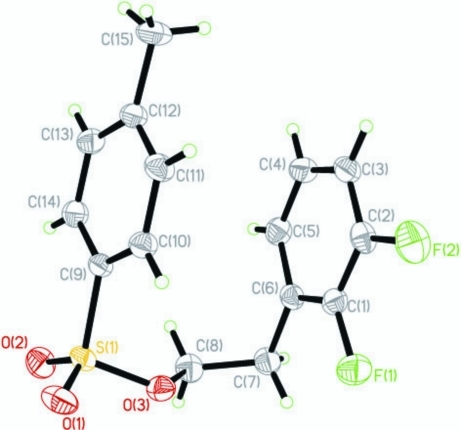
The molecular structure of (I) with displacement ellipsoids drawn at the 30% probability level and H atoms represented as spheres of arbitrary radius.

## supplementary crystallographic information

### Comment

Toluene-4-sulfonic acid 2-(2,3-difluoro-phenyl)-ethyl ester is an important intermediate for the synthesis of natural products. We have already synthesized and reported several related structures (Zhang *et al.*,2008; Xi *et al.*,2008; Wang *et al.*2008). In this research we report the X-ray crystal structure of the title compound, (I).

In the structure, the dihedral angle between the benzene(C1—C6) and benzene(C9—C14) ring is 6.18°. Weak intermolecular C–H···O hydrogen bonds and C–F···F interactions contribute to the crystal packing.

### Experimental

A solution of 2-(2,3-difluoro-phenyl)-ethanol (5 g, 32 mmol) in pyridine (15 ml) was added slowly (in 1 h) to a solution of *p*-toluenesulfonyl chloride (7.23 g, 38 mmol) in pyridine (17 ml) in ice bath. After being stirred for 3 h in ice bath, The solvent was evaporated on a rotary evaporator and the resulting solid was recrystallized in methanol, yielding the title compound (7.5 g, 76%).  Colourless blocks of (I) were grown in methanol by slow evaporation at room temperature.

### Refinement

All H atoms were fixed geometrically and treated as riding with C—H = 0.93 Å, 0.96 Å or 0.97 Å, and *U*_iso_(*H*)=1.2Ueq(*C*-methylene,*C*-aromatic) or 1.5Ueq(*C*-methyl).

### Crystal data

**Table:**

C_15_H_14_F_2_O_3_S	*Z* = 2
*M**_r_* = 312.32	*F*(000) = 324
Triclinic, *P*1	*D*_x_ = 1.363 Mg m^−^^3^
Hall symbol: -P 1	Mo *K*α radiation, λ = 0.71073 Å
*a* = 7.487 (12) Å	Cell parameters from 2108 reflections
*b* = 8.386 (14) Å	θ = 2.5–26.9°
*c* = 12.69 (2) Å	µ = 0.24 mm^−^^1^
α = 91.67 (3)°	*T* = 295 K
β = 96.51 (3)°	Block, colorless
γ = 105.65 (3)°	0.21 × 0.21 × 0.16 mm
*V* = 761 (2) Å^3^	

### Data collection

**Table:**

Bruker APEXII CCD diffractometer	2630 independent reflections
Radiation source: fine-focus sealed tube	2246 reflections with *I* > 2σ(*I*)
graphite	*R*_int_ = 0.018
φ and ω scans	θ_max_ = 25.0°, θ_min_ = 2.5°
Absorption correction: multi-scan (*SADABS*; Bruker, 2003)	*h* = −8→8
*T*_min_ = 0.955, *T*_max_ = 0.966	*k* = −9→7
4133 measured reflections	*l* = −14→15

### Refinement

**Table:**

Refinement on *F*^2^	Primary atom site location: structure-invariant direct methods
Least-squares matrix: full	Secondary atom site location: difference Fourier map
*R*[*F*^2^ > 2σ(*F*^2^)] = 0.046	Hydrogen site location: inferred from neighbouring sites
*wR*(*F*^2^) = 0.180	H-atom parameters constrained
*S* = 1.10	*w* = 1/[σ^2^(*F*_o_^2^) + (0.125*P*)^2^ + 0.1095*P*] where *P* = (*F*_o_^2^ + 2*F*_c_^2^)/3
2630 reflections	(Δ/σ)_max_ < 0.001
191 parameters	Δρ_max_ = 0.35 e Å^−^^3^
0 restraints	Δρ_min_ = −0.25 e Å^−^^3^

### Special details

**Table:**

Geometry. All e.s.d.'s (except the e.s.d. in the dihedral angle between two l.s. planes) are estimated using the full covariance matrix. The cell e.s.d.'s are taken into account individually in the estimation of e.s.d.'s in distances, angles and torsion angles; correlations between e.s.d.'s in cell parameters are only used when they are defined by crystal symmetry. An approximate (isotropic) treatment of cell e.s.d.'s is used for estimating e.s.d.'s involving l.s. planes.
Refinement. Refinement of *F*^2^ against ALL reflections. The weighted *R*-factor *wR* and goodness of fit *S* are based on *F*^2^, conventional *R*-factors *R* are based on *F*, with *F* set to zero for negative *F*^2^. The threshold expression of *F*^2^ > σ(*F*^2^) is used only for calculating *R*-factors(gt) *etc*. and is not relevant to the choice of reflections for refinement. *R*-factors based on *F*^2^ are statistically about twice as large as those based on *F*, and *R*- factors based on ALL data will be even larger.

### Fractional atomic coordinates and isotropic or equivalent isotropic displacement parameters (Å^2^)

**Table:**

	*x*	*y*	*z*	*U*_iso_*/*U*_eq_	
S1	1.10764 (8)	0.91567 (7)	0.72239 (5)	0.0436 (3)	
F1	0.7545 (3)	0.2961 (2)	1.01476 (15)	0.0728 (6)	
F2	0.8697 (2)	0.6359 (2)	1.04849 (14)	0.0664 (5)	
O1	1.3018 (3)	0.9177 (3)	0.75887 (18)	0.0631 (6)	
O2	1.0724 (3)	1.0423 (2)	0.65329 (16)	0.0631 (6)	
O3	1.0172 (2)	0.9271 (2)	0.83135 (14)	0.0489 (5)	
C1	0.5485 (4)	0.3112 (3)	0.8590 (2)	0.0493 (6)	
H1	0.5108	0.1962	0.8487	0.059*	
C2	0.6824 (4)	0.3906 (3)	0.9445 (2)	0.0474 (6)	
C3	0.7407 (3)	0.5666 (3)	0.96112 (19)	0.0429 (6)	
C4	0.6655 (3)	0.6682 (3)	0.8921 (2)	0.0430 (6)	
C5	0.5301 (4)	0.5870 (3)	0.8060 (2)	0.0498 (6)	
H5	0.4780	0.6508	0.7597	0.060*	
C6	0.4721 (4)	0.4123 (4)	0.7884 (2)	0.0542 (7)	
H6	0.3841	0.3633	0.7308	0.065*	
C7	0.7222 (4)	0.8606 (3)	0.9135 (2)	0.0525 (7)	
H7A	0.8020	0.8899	0.9808	0.063*	
H7B	0.6105	0.8961	0.9195	0.063*	
C8	0.8273 (4)	0.9564 (3)	0.8242 (2)	0.0532 (7)	
H8A	0.7579	0.9164	0.7549	0.064*	
H8B	0.8392	1.0741	0.8340	0.064*	
C9	0.9802 (3)	0.7116 (3)	0.66443 (19)	0.0369 (5)	
C10	1.0027 (4)	0.5709 (3)	0.7179 (2)	0.0430 (6)	
H10	1.0835	0.5837	0.7808	0.052*	
C11	0.9002 (4)	0.4104 (3)	0.6741 (2)	0.0479 (6)	
H11	0.9161	0.3180	0.7086	0.057*	
C12	0.7747 (3)	0.3873 (3)	0.5795 (2)	0.0469 (6)	
C13	0.7558 (4)	0.5309 (3)	0.5248 (2)	0.0511 (7)	
H13	0.6763	0.5179	0.4614	0.061*	
C14	0.8594 (4)	0.6939 (3)	0.5678 (2)	0.0466 (6)	
H14	0.8475	0.7870	0.5327	0.056*	
C15	0.6567 (5)	0.2110 (4)	0.5350 (3)	0.0740 (10)	
H15A	0.7362	0.1383	0.5346	0.111*	
H15B	0.6007	0.2168	0.4638	0.111*	
H15C	0.5605	0.1691	0.5791	0.111*	

### Atomic displacement parameters (Å^2^)

**Table:**

	*U*^11^	*U*^22^	*U*^33^	*U*^12^	*U*^13^	*U*^23^
S1	0.0463 (4)	0.0295 (4)	0.0505 (4)	0.0020 (3)	0.0082 (3)	0.0014 (3)
F1	0.0742 (12)	0.0693 (12)	0.0839 (13)	0.0319 (10)	0.0117 (10)	0.0265 (10)
F2	0.0549 (10)	0.0767 (12)	0.0594 (11)	0.0096 (8)	−0.0050 (8)	−0.0008 (8)
O1	0.0448 (11)	0.0510 (11)	0.0832 (15)	−0.0004 (8)	0.0024 (10)	−0.0082 (10)
O2	0.0868 (15)	0.0357 (10)	0.0659 (13)	0.0110 (10)	0.0173 (11)	0.0143 (9)
O3	0.0545 (11)	0.0423 (10)	0.0471 (11)	0.0101 (8)	0.0038 (8)	−0.0037 (8)
C1	0.0527 (15)	0.0376 (13)	0.0559 (16)	0.0057 (11)	0.0167 (12)	−0.0006 (11)
C2	0.0452 (14)	0.0464 (14)	0.0560 (16)	0.0165 (11)	0.0175 (12)	0.0132 (12)
C3	0.0356 (12)	0.0515 (15)	0.0402 (13)	0.0084 (10)	0.0077 (10)	−0.0008 (11)
C4	0.0425 (13)	0.0397 (14)	0.0476 (14)	0.0089 (11)	0.0146 (11)	0.0023 (10)
C5	0.0528 (15)	0.0511 (15)	0.0471 (15)	0.0172 (12)	0.0047 (11)	0.0066 (12)
C6	0.0501 (15)	0.0521 (16)	0.0532 (16)	0.0031 (12)	0.0050 (12)	−0.0047 (13)
C7	0.0617 (16)	0.0400 (14)	0.0580 (16)	0.0141 (12)	0.0180 (13)	−0.0007 (12)
C8	0.0637 (17)	0.0395 (14)	0.0618 (17)	0.0203 (12)	0.0148 (13)	0.0060 (12)
C9	0.0366 (12)	0.0320 (12)	0.0403 (12)	0.0058 (9)	0.0061 (10)	0.0011 (9)
C10	0.0458 (13)	0.0388 (13)	0.0423 (13)	0.0100 (10)	0.0011 (10)	0.0026 (10)
C11	0.0543 (15)	0.0329 (13)	0.0562 (16)	0.0106 (11)	0.0088 (12)	0.0051 (11)
C12	0.0420 (13)	0.0414 (14)	0.0531 (15)	0.0025 (11)	0.0133 (11)	−0.0085 (11)
C13	0.0456 (14)	0.0582 (16)	0.0414 (14)	0.0036 (12)	−0.0004 (11)	−0.0042 (12)
C14	0.0523 (14)	0.0442 (14)	0.0415 (13)	0.0109 (11)	0.0033 (11)	0.0076 (10)
C15	0.072 (2)	0.0494 (17)	0.084 (2)	−0.0101 (15)	0.0149 (17)	−0.0241 (16)

### Geometric parameters (Å, °)

**Table:**

S1—O2	1.456 (3)	C7—H7A	0.9700
S1—O1	1.470 (3)	C7—H7B	0.9700
S1—O3	1.618 (3)	C8—H8A	0.9700
S1—C9	1.808 (3)	C8—H8B	0.9700
F1—C2	1.369 (3)	C9—C14	1.417 (4)
F2—C3	1.384 (3)	C9—C10	1.417 (4)
O3—C8	1.500 (4)	C10—C11	1.418 (4)
C1—C2	1.406 (4)	C10—H10	0.9300
C1—C6	1.428 (4)	C11—C12	1.412 (4)
C1—H1	0.9300	C11—H11	0.9300
C2—C3	1.423 (5)	C12—C13	1.438 (4)
C3—C4	1.414 (4)	C12—C15	1.555 (4)
C4—C5	1.421 (4)	C13—C14	1.434 (4)
C4—C7	1.561 (4)	C13—H13	0.9300
C5—C6	1.414 (5)	C14—H14	0.9300
C5—H5	0.9300	C15—H15A	0.9600
C6—H6	0.9300	C15—H15B	0.9600
C7—C8	1.569 (4)	C15—H15C	0.9600
			
O2—S1—O1	119.16 (13)	O3—C8—C7	108.0 (2)
O2—S1—O3	109.80 (14)	O3—C8—H8A	110.1
O1—S1—O3	103.90 (15)	C7—C8—H8A	110.1
O2—S1—C9	110.16 (16)	O3—C8—H8B	110.1
O1—S1—C9	109.48 (13)	C7—C8—H8B	110.1
O3—S1—C9	103.00 (12)	H8A—C8—H8B	108.4
C8—O3—S1	118.26 (17)	C14—C9—C10	121.1 (2)
C2—C1—C6	118.1 (3)	C14—C9—S1	120.24 (19)
C2—C1—H1	121.0	C10—C9—S1	118.7 (2)
C6—C1—H1	121.0	C9—C10—C11	119.0 (2)
F1—C2—C1	119.1 (3)	C9—C10—H10	120.5
F1—C2—C3	119.5 (3)	C11—C10—H10	120.5
C1—C2—C3	121.4 (2)	C12—C11—C10	121.8 (2)
F2—C3—C4	120.7 (3)	C12—C11—H11	119.1
F2—C3—C2	118.2 (2)	C10—C11—H11	119.1
C4—C3—C2	121.1 (3)	C11—C12—C13	118.7 (2)
C3—C4—C5	117.1 (3)	C11—C12—C15	121.2 (3)
C3—C4—C7	121.1 (2)	C13—C12—C15	120.1 (3)
C5—C4—C7	121.7 (2)	C14—C13—C12	120.2 (3)
C6—C5—C4	122.2 (3)	C14—C13—H13	119.9
C6—C5—H5	118.9	C12—C13—H13	119.9
C4—C5—H5	118.9	C9—C14—C13	119.2 (2)
C5—C6—C1	120.1 (3)	C9—C14—H14	120.4
C5—C6—H6	120.0	C13—C14—H14	120.4
C1—C6—H6	120.0	C12—C15—H15A	109.5
C4—C7—C8	113.2 (2)	C12—C15—H15B	109.5
C4—C7—H7A	108.9	H15A—C15—H15B	109.5
C8—C7—H7A	108.9	C12—C15—H15C	109.5
C4—C7—H7B	108.9	H15A—C15—H15C	109.5
C8—C7—H7B	108.9	H15B—C15—H15C	109.5
H7A—C7—H7B	107.7		
			
O2—S1—O3—C8	41.5 (2)	S1—O3—C8—C7	146.04 (19)
O1—S1—O3—C8	170.02 (17)	C4—C7—C8—O3	−70.1 (3)
C9—S1—O3—C8	−75.8 (2)	O2—S1—C9—C14	−3.1 (2)
C6—C1—C2—F1	178.5 (2)	O1—S1—C9—C14	−136.0 (2)
C6—C1—C2—C3	0.1 (4)	O3—S1—C9—C14	114.0 (2)
F1—C2—C3—F2	0.1 (3)	O2—S1—C9—C10	177.34 (18)
C1—C2—C3—F2	178.4 (2)	O1—S1—C9—C10	44.5 (2)
F1—C2—C3—C4	−178.1 (2)	O3—S1—C9—C10	−65.6 (2)
C1—C2—C3—C4	0.2 (4)	C14—C9—C10—C11	−0.9 (4)
F2—C3—C4—C5	−178.3 (2)	S1—C9—C10—C11	178.70 (19)
C2—C3—C4—C5	−0.1 (3)	C9—C10—C11—C12	−0.9 (4)
F2—C3—C4—C7	−1.2 (3)	C10—C11—C12—C13	2.1 (4)
C2—C3—C4—C7	177.0 (2)	C10—C11—C12—C15	−177.3 (2)
C3—C4—C5—C6	−0.3 (4)	C11—C12—C13—C14	−1.7 (4)
C7—C4—C5—C6	−177.4 (2)	C15—C12—C13—C14	177.7 (2)
C4—C5—C6—C1	0.6 (4)	C10—C9—C14—C13	1.2 (4)
C2—C1—C6—C5	−0.5 (4)	S1—C9—C14—C13	−178.31 (18)
C3—C4—C7—C8	116.4 (3)	C12—C13—C14—C9	0.0 (4)
C5—C4—C7—C8	−66.7 (3)		

### Hydrogen-bond geometry (Å, °)

**Table:**

*D*—H···*A*	*D*—H	H···*A*	*D*···*A*	*D*—H···*A*
C1—H1···O1^i^	0.93	2.58	3.442 (7)	154

Symmetry codes: (i) *x*−1, *y*−1, *z*.
